# Testing Messages on Facebook to Promote Use of an HPV Educational Web-Intervention

**DOI:** 10.3389/fdgth.2021.648555

**Published:** 2021-03-12

**Authors:** Jenna E. Reno, Amanda F. Dempsey

**Affiliations:** ^1^Department of Family Medicine, School of Medicine, University of Colorado Denver, Aurora, CO, United States; ^2^Adult and Child Consortium for Health Outcomes Research and Delivery Science, University of Colorado Denver, Aurora, CO, United States

**Keywords:** HPV vaccination, vaccine hesitancy, fear appeals, persuasion, health communication, social media, dissemination & implementation research

## Abstract

In the US, the human papillomavirus (HPV) vaccine remains underutilized leading to disparities in HPV-related diseases. Latinx have some of the highest rates of cancer caused by HPV. In a previous study, we developed a tailored-messaging based online educational intervention (CHICOS) that was found to increase HPV vaccination intention among Latinx participants. The current research uses Facebook Advertising to test the comparative effectiveness of messages designed using the Extended Parallel Processing Model (EPPM) to promote the use of CHICOS among Latinx young adults and parents of adolescents. We also looked at differences in the effectiveness of messages that highlighted HPV-related cancers, genital warts, or a control condition as well as differences in Spanish vs. English messages. Results found Latinx young adults and parents, were more likely to click on Facebook Advertisements containing messages in Spanish and those that mention cancer risks pertinent to this population compared to those in English or messages that discuss genital warts. Thus, findings suggest that Facebook Advertising has the potential to be a useful tool for motivating information seeking online about HPV vaccination.

## Introduction

Human papillomavirus (HPV) is the most common sexually transmitted infection worldwide (1). Latinx have higher rates of HPV-related cancers compared to other groups in the United States (1). Previous research demonstrates the efficacy of health communication efforts that are customized to include culturally relevant information (2–5). The current study presents a comparison of health messages aimed at promoting HPV information seeking as a means for encouraging HPV vaccination among a Latinx audience.

The burden of HPV infection exacts a significant emotional, financial, and medical toll (6, 7). Although rates of HPV infection are similar across nearly all race and ethnicity groups, there are significant disparities in HPV-associated cancers among Latinx. For example, Latinas have the highest risk of developing cervical cancer when compared to all other US population groups—similarly, Latinos have the highest risk of developing penile cancer (1, 7–10). Additionally, Latinx experience higher rates of other HPV-related illnesses like genital warts, abnormal Pap smears, anal cancer, and oropharyngeal cancer (6).

Although a vaccine for HPV has been available and recommended by the Centers for Disease Control and Prevention (CDC) for the past 10 years for routine use among all adolescents ages 11 years and older, US vaccination rates continue to remain low (11). As of 2019, it is estimated that only 56.8% of girls and 51.8% of boys ages 13–17 nationally were up-to-date on the HPV vaccine series (11). Vaccination series completion rates are even lower among Latinx adolescents (46.2% for girls, 35.0% for boys) and significantly below the national vaccination target level of 80% coverage (12). Without significant increases in HPV vaccination, especially among high-risk populations, disparities in HPV-related cancers and other diseases are likely to continue. Thus, there is an urgent need for interventions that employ culturally-targeted strategies to reduce these disparities.

In a previous study, we developed a web-based educational intervention targeting Latinx parents of adolescents and young adults, called CHiCOS (13). CHiCOS uses responses to a series of survey items to provide personally tailored information about HPV. Results from a randomized controlled study comparing CHiCOS to “usual care” demonstrated that exposure to CHiCOS led to increased intention to vaccinate for HPV. These increased intentions unfortunately did not lead to subsequent increases in vaccination receipt. Later analysis indicated that this lack of effect was most likely due to logistical barriers to receiving the vaccine. Given that positive intentions are recognized as a necessary upstream factor to successful vaccination (14–19), the CHICOS intervention can be added to the growing list of evidence based communication strategies to improve vaccination intention.

Thus, CHiCOS presents the opportunity to further explore methods for the dissemination of evidence-based communication interventions which increase HPV vaccination intent. Previous attempts to disseminate similar web-based interventions in healthcare office waiting rooms have produced lack-luster results (13)—demonstrating the need to discover alternative methods for disseminating CHiCOS in a manner that stimulates the health information seeking behavior necessary to engage with the website. Health information seeking is particularly relevant as previous research has demonstrated its role in promoting HPV vaccine intentions (20–22). For the purposes of the current study, the primary outcome of interest is to stimulate use of the CHiCOS website—a form of health information seeking specific to HPV vaccination—via social media advertising. As such, the primary aim of the current study is to compare the effectiveness of different messages for promoting HPV information seeking via CHiCOS.

The Extended Parallel Process Model [EPPM; (23)] is a theoretical framework for designing messages that has previously proven successful for promoting HPV vaccination (24, 25). The EPPM posits that message design strategies that highlight risk *severity* and the target audiences' *susceptibility* must be coupled with messages prompting *self-efficacy* and *response efficacy* in order to effectively persuade individuals to take protective action (26). As people may underestimate their susceptibility to and the severity of HPV-related diseases (27–31)—especially cancers outside of cervical cancer including those that affect men (i.e., anal, penile, and oropharyngeal cancers)—the EPPM is a particularly relevant theoretical framework for designing messages to promote HPV vaccination. Previous research on using the EPPM to design messages related to HPV vaccination has primarily focused on intention to vaccinate as the primary outcome (32). However, other work has demonstrated the utility of the EPPM for promoting health information seeking in areas such as meningitis vaccination (33).

Results of previous studies demonstrate that message content (cervical cancer vs. genital warts) may differently influence parents' vs. young adults' intention to vaccinate [cf. (25)]. However, these messages have not been explicitly tested among a Latinx population—a group with distinct cultural norms and values related to sexual health and healthcare decision making. The current study extends this line of research. We posit the following hypotheses and research questions:

H1: Among Latinx parents of HPV-vaccination eligible adolescents, messages focusing on HPV-related cancers will lead to higher information seeking via CHICOS (i.e., link click–through rates) than messages focusing on genital warts.
H2: Among Latinx young adults, messages focusing on genital warts will lead to higher information seeking via CHICOS (i.e., link click–through rates) than messages focusing on HPV-related cancers.
RQ1: Do messages containing EPPM variables lead to higher click-through rates than messages without these variables?
RQ2: Does message language affect click-through rates?

## Materials and Methods

**Figure 1:**
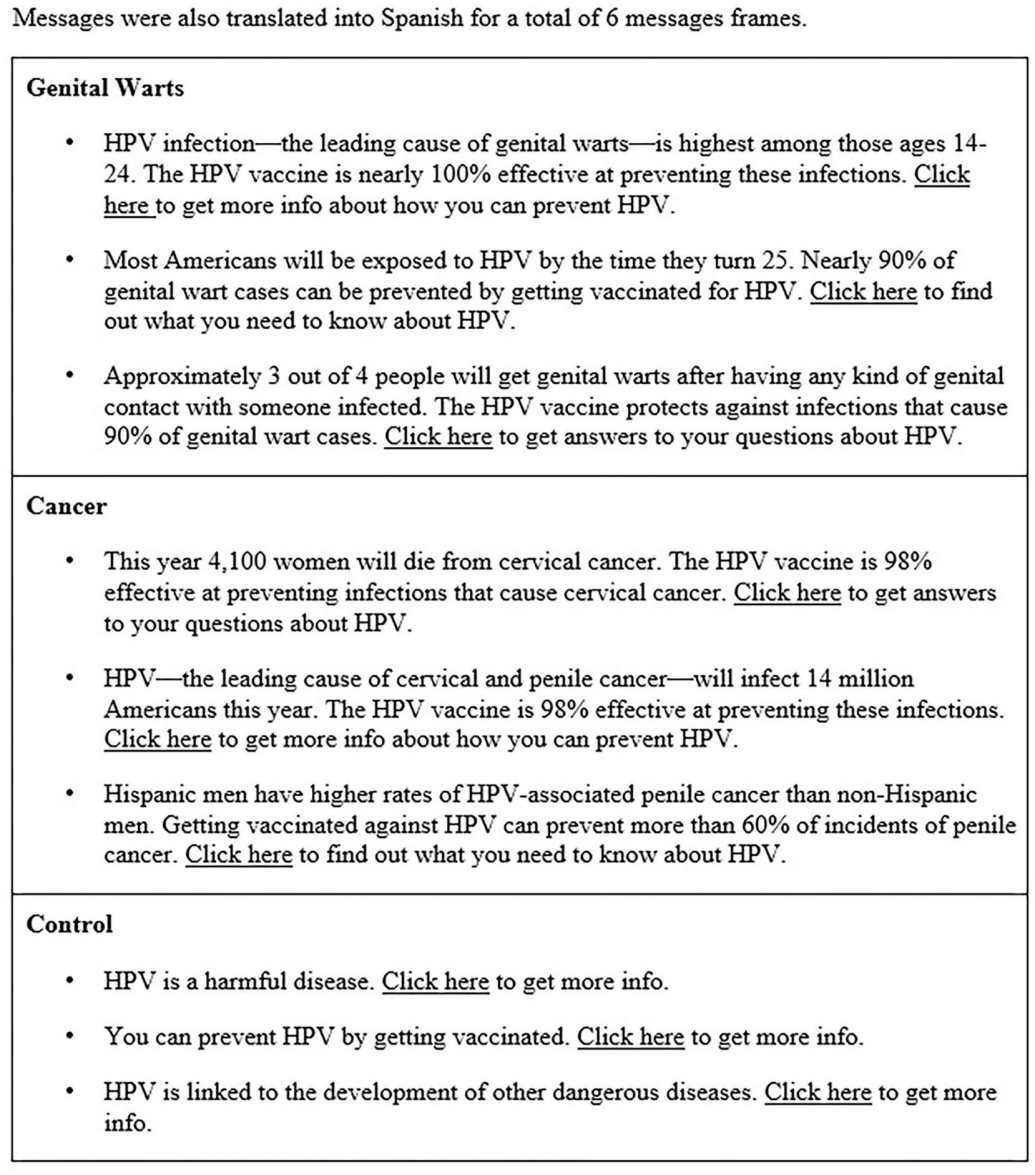
Message frames.

The current study compared message effectiveness for promoting use and dissemination of CHiCOS, a web-based educational intervention [previously described in detail, see (13, 34)]. We used Facebook Advertising to test nine messages designed using the EPPM (see Figure 1) and employed a 2 × 3 × 2 mixed factorial design that compared two key decision-making audiences (Latinx young adults vs. parents of adolescents), three message topic frames (genital warts vs. cancer vs. control), and two message languages (English vs. Spanish). Per the EPPM, messages in the cancer and genital warts frames included *susceptibility* and *severity* information specific to Latinx populations, as well as *response efficacy* statements emphasizing the effectiveness of the HPV vaccine. *Self-efficacy* for seeking information about the HPV vaccine was induced by directing users to a link where more information about HPV could be found (i.e., the CHiCOS website). Messages in the control frame were written to be intentionally vague, did not mention cancer or genital warts, and did not meet criteria for containing all EPPM message components.

**Figure 2:**
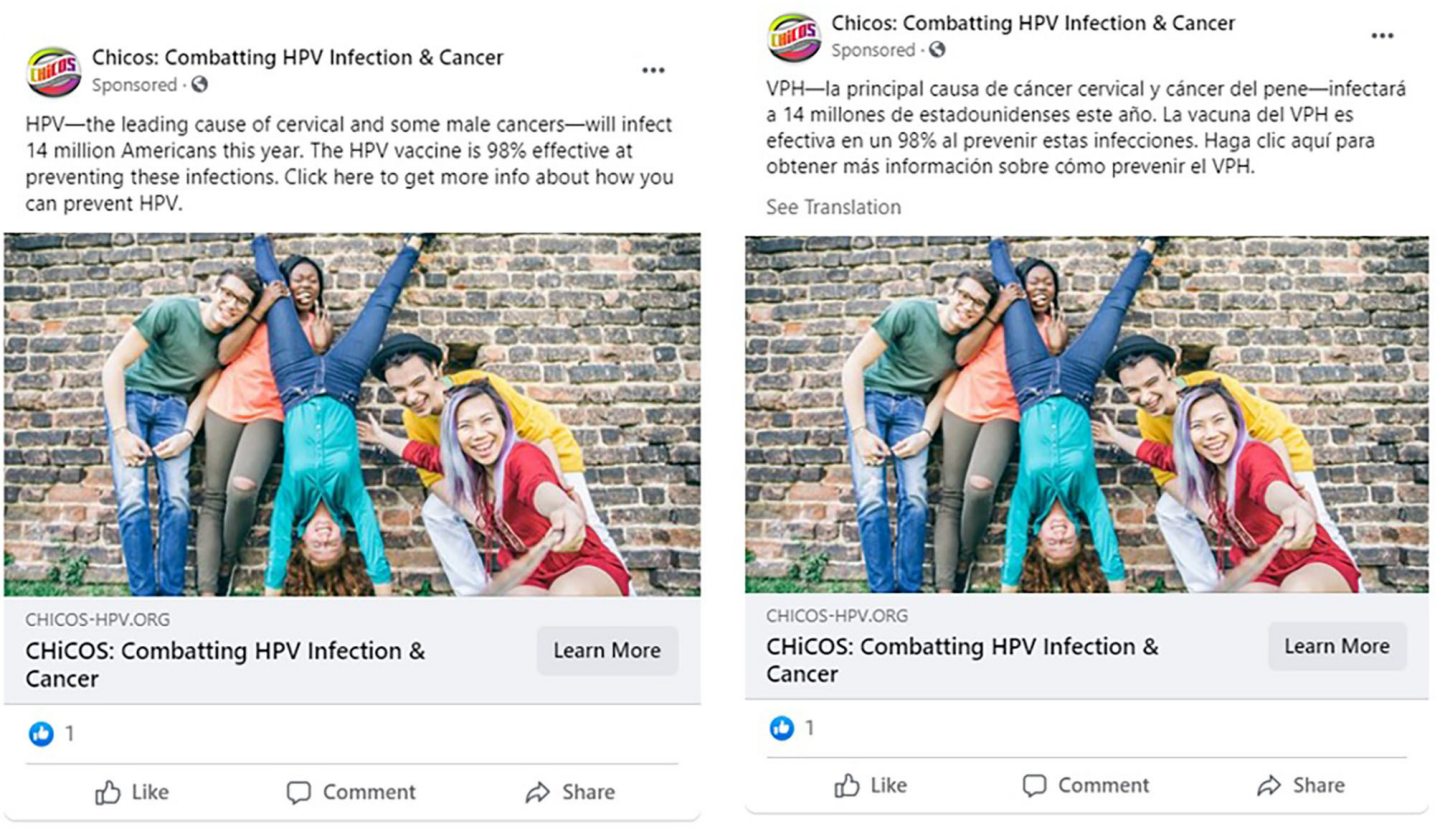
Example of facebook advertisements.

Facebook Advertising was chosen as the method of study for its popularity and ability to reach target audience members, the capacity to embed website links to the CHiCOS website in advertising messages, and Facebook's role in facilitating health information seeking (35, 36). Advertisement sets were created for each message frame containing three advertisements, each with a different message from the frame (see Figure 1). All advertisements used the same stock photo of multicultural youth. To comply with Facebook's requirements for establishing a source account to sponsor advertisements, we created a Facebook Page with information about the CHiCOS website. The account for this page was then used as the sponsoring source for all advertisements (see Figure 2). Facebook advertisements ran for 39 consecutive days in October and November 2017.

### Subjects

Facebook's advertising settings provided the ability to target different population groups for the study. Messages were targeted to Latinx young adults using the Facebook Advertisement criteria: Ages 18–27, Behaviors: Multicultural Affinity: Hispanic (US - All), and geographically relevant to the study team (i.e., Denver metro area) zip codes with high Latinx populations. Similarly, Latinx parents of adolescents were targeted using the criteria: Ages 28–65 (to exclude overlap with young adult advertising sets), Behaviors: Multicultural Affinity: Hispanic (US - All), Parents, and Denver metro area zip codes with high Latinx populations. Different zip codes were used for each message frame advertising set (see Figure 1) so that users did not overlap between the six message conditions. Using population data for each zip code, we created advertising sets that were estimated to reach 18,000–19,000 unique users each.

### Data Sources

Data were collected using Facebook analytics, Google analytics, and CHiCOS website paradata in order to determine the comparative effectiveness of message frames based on each advertisement's performance metrics. The primary outcome of interest was click-through rates to the CHiCOS website—wherein, for the purposes of our study, information seeking behavior was defined as clicking on a link embedded in each advertisement that led to the CHiCOS website.

#### Facebook Analytics

Metrics provided by Facebook included: (1) Reach - the number of unique users who saw the ad at least once, (2) Impressions – the number of total times the ad was on screen for the targeted audience, (3) Frequency - the average number of times each user saw the ad, (4) Link clicks – the number of clicks on a link within the ad that led to the CHiCOS website, and (5) Link click-through rate (CTR) - the percentage of times people saw the ad and performed a link click (i.e., link clicks/impressions), (6) Unique link clicks – the number of unique users who clicked on the website link (i.e., removes duplicate link clicks by the same user), and (7) Unique CTR – the percentage of unique users who saw the ad and performed a link click (37).

Since Facebook uses a proprietary algorithm to determine the priority by which it places advertisements in users' timelines, parameters for controlling the reach of ads were not entirely within our control. Our goal was for each ad set to reach at least 3,000 users in order to compare performance of our primary outcome (Unique CTR). However, Facebook does not allow advertisers to place limits on Reach – only on the total amount of money spent for each ad. Thus, for the purposes of this study, Reach serves as an outcome that reflects ad performance as the algorithm adjusts to extend the reach of certain ads that produce more results (e.g., higher engagement) early on and to limit the reach of ads that produce lower results. This algorithm frequently changes and Facebook does not disclose the exact parameters they use to extend or limit ad reach.

#### Google Analytics

Metrics provided by Google analytics (which was installed on the CHiCOS website) included: (1) Sessions - the period time a user is actively engaged with the website, (2) Pageviews – the total number of web pages viewed on the website, (3) Avg. session duration - the average time length of a session, (4) Page depth - the average number of pages viewed during a session, (5) Bounce rate – percentage of sessions with only one page view out of all sessions (38). Google analytics tracked the website referral source (Facebook) using unique referral links embedded into different advertising message frames. Thus, we were able to track the above metrics for each message frame.

#### CHiCOS Website Para-Data

The CHiCOS website reported metrics on the total number of time spent on the website (for users who registered to use the site by clicking the continue button on the landing page), as well as whether or not (True/False) the user completed the survey items used to tailor the educational messaging.

### Analysis

Descriptive statistics as well as chi-square tests for categorical data, and z-tests and g-tests for proportions, were used to examine differences between groups (H1-2, RQ 1-2).

## Results

### Facebook Analytics

Overall, messages reached 73,081 Facebook users and generated 238,365 impressions for a frequency of 3.23 impressions per user. Table 1 provides a breakdown of all reach, impression, and frequency data by message frame for parents and young adults.

**Table 1:** Facebook analytics for reach, impressions, and frequency by message frame.

**Message frame**	**Reach**	**Impressions**	**Frequency**	**X^**2**^(*p*)**
**Parents**				171.16 (<0.001)
Cancer English	4,066	11,101	2.73	
Cancer Spanish	5,465	19,807	3.62	
Control English	4,099	10,672	2.60	
Control Spanish	5,654	18,005	3.18	
Genital Warts English	6,316	18,089	2.86	
Genital Warts Spanish	6,375	17,930	2.81	
**Young adults**				52.87 (*p* < 0.001)
Cancer English	6,338	25,079	3.96	
Cancer Spanish	6,157	23,480	3.81	
Control English	6,650	22,993	3.46	
Control Spanish	7,719	23,810	3.08	
Genital Warts English	6,894	23,602	3.42	
Genital Warts Spanish	7,348	23,797	3.24	
**TOTAL (Reach, Impressions)**	73,081	238,365		
**AVG. (Frequency)**			3.23	

A chi-square test of independence was performed to examine the relation between language and message frame. For both parents and young adults, the relationship between these variables was significant [parents: X2 (1, *N* = 31,975) = 171.16, *p* < 0.001; young adults, X2 (1, *N* =41,106) = 52.87, *p* < 0.001.). Across both parents and young adults, advertisements in Spanish reached more users than messages in English—and messages that mentioned genital warts reached more users than messages about cancer or the control condition.

Overall, Facebook advertisements produced 2,159 total link clicks and 1,552 unique link clicks to the CHiCOS website across all message conditions. The overall Unique CTR was 0.9% (per impression) and the unique CTR was 2.12% (per user reached). Table 2 provides a breakdown of link clicks and CTR by message frame for parents and young adults.

**Table 2:** Facebook analytics for link clicks and CTR by message frame.

**Campaign name**	**Link clicks**	**CTR (link click-through rate)**	**Unique link clicks**	**Unique CTR (link click-through rate)**	**G (*p*)**	**z (*p*)**
**Parents**						
Cancer English	91	0.82	77	1.89		
Cancer Spanish	279	1.41	169	3.09		
Control English	77	0.72	69	1.68		
Control Spanish	155	0.86	119	2.10		
Genital Warts English	155	0.86	119	1.88		
Genital Warts Spanish	166	0.93	123	1.93		
Cancer Avg.				2.49	13.4	
Control Avg.				1.89	(0.001)	
Genital Warts Avg.				1.91		
English Avg.				2.35		0.16
Spanish Avg.				1.82		(0.001)
**Young adults**						
Cancer English	201	0.80	158	2.49		
Cancer Spanish	267	1.14	161	2.61		
Control English	166	0.72	128	1.92		
Control Spanish	205	0.86	150	1.94		
Genital Warts English	204	0.86	140	2.03		
Genital Warts Spanish	193	0.81	139	1.89		
Cancer Avg.					14.5	
Control Avg.					(0.001)	
Genital Warts Avg.						
English Avg.						0.16
Spanish Avg.						(0.873)
**TOTAL (Clicks)**	2,159		1,552.00			
**AVG (CTR)**		0.90		2.12		

For Latinx parents, messages reached 31,975 users and generated 676 Unique link clicks for an average Unique CTR of 2.10%. Messages mentioning cancer had a significantly higher Unique CTR (2.49) than control (1.89) and genital warts message (1.91, *p* = 0.001)—thus, our hypothesis that cancer messages would produce higher CTR among Latinx parents (H1) was confirmed. Spanish messages had a higher overall Unique CTR (2.35) than English messages (*p* = 0.001, RQ2).

For Latinx young adults, messages reached 41,106 users and generated 876 Unique link clicks for an average Unique CTR of 2.15%. Messages mentioning cancer had a significantly higher Unique CTR (2.55) than control (1.93) and genital warts messages (1.96, *p* = 0.001)— disconfirming our hypothesis that genital wart messages would produce higher CTR among Latinx young adults (H2). There were no significant differences in the overall Unique CTR for English (2.14%) vs. Spanish messages (2.12%).

### Google Analytics

Session metrics from Google Analytics demonstrated similar numbers for total new visitor website sessions (1,592; with confirmed referrals from Facebook) when compared to Facebook Analytics' Unique link clicks (1,552). Table 3 provides a full breakdown of Google Analytic metrics by message frame for parents and young adults. Across both user groups, the bounce rate was high, 92.58%–indicating that the majority of users did not proceed past the first page of the website. For those that did proceed past the first page of the website, the average session duration was 24.64 seconds with a page depth average of 1.21 pages per user session.

**Table 3:** Google analytics data.

**Shared URL**	**Sessions**	**Pageviews**	**Avg. session duration**	**Page depth**
**Parents**				
Cancer English	100	118	5.44	1.24
Cancer Spanish	232	284	10.92	1.23
Control English	74	81	2.72	1.08
Control Spanish	125	161	60.37	1.31
Genital Warts English	144	162	12.76	1.11
Genital Warts Spanish	151	197	24.68	1.28
**Young adults**				
Cancer English	177	197	47.85	1.11
Cancer Spanish	241	285	44.63	1.20
Control English	143	182	19.18	1.26
Control Spanish	217	264	18.24	1.24
Genital Warts English	182	229	34.31	1.26
Genital Warts Spanish	173	204	14.58	1.20
**Total (Sessions, Pageviews), Avg. (Session duration, Page depth)**	1,959^*^	2,364	24.64	1.21

* *This number is based on direct referrals from Facebook. Google Analytics showed an additional 200 sessions during this time that may be the result of users navigating to the CHiCOS website from Facebook through other pathways instead of directly clicking the link within Facebook advertisements. Thus, they were not counted here as they did not use the unique links we embedded in advertisements to track differences in message frame performance*.

Additionally, Google's analytics showed that, in addition to the 1,592 new visitor sessions, 261 users returned the CHiCOS website at least once generating 565 additional sessions (for a total of 2,157 sessions). The bounce rate was similarly high among returning users (92.21%); however, average session duration was considerably longer at 53.01 seconds.

### CHiCOS Website Para-Data

While Facebook advertising messages produced 1,552 link clicks to the website, paradata tracking new users to the website showed that only 11 people interacted with the website—and of those, only three people completed the survey items necessary to receive the personally tailored educational materials. The average time among those who interacted with the website was 8:52 minutes.

## Discussion

The current study provides evidence that using Facebook advertising was an effective means for promoting information seeking about HPV vaccination via the CHiCOS website. Overall, messages in Spanish were more effective at reaching Latinx young adults and parents of adolescents than were messages in English. Among Latinx parents, messages that mentioned the relationship between HPV and cancer produced higher proportions of information seeking behavior (i.e., unique CTR) than messages that referenced genital warts or the control messages. However, among Latinx young adults, we did not find the reverse trend (as we hypothesized in H2)—that is, messages mentioning genital warts did not generate higher unique CTR than messages about cancer or the control messages.

Messages that contained all four elements as prescribed by the EPPM did not consistently outperform messages without (i.e., the control message frame). This finding provides further support for results of a previous message testing study where survey participants were shown the same messages used here containing a hyperlink to “more information” (Reno and Dempsey, unpublished manuscript). Alternatively, participants could choose to continue on in the survey without clicking the hyperlink. In this study, participants who received a control message were more likely to click the hyperlink than those who received either the cancer or genital warts messaging. Furthermore, when asked to report the extent to which a message made them “feel frightened,” participants who received the control messages consistently reported higher levels of fear than those who received the cancer or genital warts messages containing statements of severity and susceptibility designed to induce fear. Thus, further research is needed to understand the role of message design in promoting information seeking, particularly about HPV vaccination. It may be that messages that present a more veiled threat (as opposed to containing explicit susceptibility and severity statements) are more likely to inspire information seeking behavior.

We had 261 users total who visited the website more than once, demonstrating that messages disseminated via Facebook advertising were effective at stimulating initial information seeking behavior (i.e., clicking on a link for more information). However, our study did not produce evidence this information seeking behavior was carried out once users reached the CHiCOS website, as demonstrated by the high bounce rate even among repeat users—although there is some evidence that repeat users invested more time in information seeking via CHiCOS as their total time spent on the website (i.e., avg. session duration) was longer than new users. This may be due to limitations in the design of the CHiCOS website where users where first instructed to complete a short survey before receiving personalized information about the HPV vaccine. Thus, it stands to reason that users were not compelled to seek information to the extent needed to complete the survey items before receiving more information about the HPV vaccine. Further research is necessary to determine type of message content and format sufficient to engage users in HPV information seeking.

## Limitations

A primary limitation to the current work is the format of the CHiCOS website. We have inferred from the data based on the large discrepancy between those who clicked on the link to the website and those who completed the survey questions necessary to view the educational information about HPV that the design of the website may be a primary barrier to additional information seeking. However, it is also probable that the messages delivered via Facebook advertising were insufficient to inspire prolonged information seeking behavior. The design of the study is limited in its ability to substantively differentiate between these two plausible explanations. Thus, further research is needed to determine the ability of Facebook advertising to promote information seeking and dissemination of web-based interventions for HPV.

Additionally, the limitations inherent to the secrecy of the Facebook algorithm impeded our ability to control for variations in advertisement reach and impressions. Thus, while this approach provided a pragmatic test of comparative message effectiveness for promoting the dissemination and use of CHiCOS, we cannot construe the extent to which variations in reach and impressions were due to aspects of the message or of the function of the algorithm. Future research should further explore the utility of Facebook Advertising for this type of A/B message testing—a feature that has more recently been added to the Facebook Advertising platform (37).

## Conclusion

Overall, results suggest that Facebook Advertising is an effective means for disseminating and inspiring interest in web-based health information resources about HPV vaccination. As vaccine hesitancy continues to grow, it is essential to identify effective means to deliver accurate information about vaccines such as the HPV vaccine and other effective practices for promoting vaccination.

## Data Availability Statement

The raw data supporting the conclusions of this article will be made available by the authors, without undue reservation.

## Ethics Statement

The studies involving human participants were reviewed and approved by Colorado Multiple Institutional Review Board (COMIRB). Written informed consent for participation was not required for this study in accordance with the national legislation and the institutional requirements.

## Author Contributions

JR was responsible for the overall content as guarantor. Both authors contributed to the planning, conduct, and reporting of the work described in the article.

## Conflict of Interest

At the time of the study, AD was an advisory board member for Merck and Pfizer. The remaining author declares that the research was conducted in the absence of any commercial or financial relationships that could be construed as a potential conflict of interest.
